# *N*-(2-Furo­yl)-*N*′-(2-pyrid­yl)thio­urea

**DOI:** 10.1107/S1600536809011301

**Published:** 2009-03-31

**Authors:** O. Estévez-Hernández, J. Duque, H. Pérez, S. Santos Jr, Y. Mascarenhas

**Affiliations:** a Laboratory of Molecular Engineering, Institute of Materials (IMRE), University of Havana, Cuba; b Departamento de Química Inorgánica, Facultad de Química, Universidad de La Habana, Cuba; c Laboratório de Física, Universidade Federal do Tocantins, Palmas, Tocantins, Brazil; d Instituto de Física de São Carlos, Universidade de São Paulo, São Carlos, Brazil

## Abstract

The title compound, C_11_H_9_N_3_O_2_S, crystallizes with two independent mol­ecules in the asymmetric unit. The central thio­urea core makes dihedral angles of −3.3 (3) and 0.6 (3)° with the furan carbonyl groups in each mol­ecule, whereas the pyridine ring is inclined by 4.63 (2) and 11.28 (7)°, respectively. The *trans*–*cis* geometry of the thio­urea fragment is stabilized by an intra­molecular N—H⋯N hydrogen bond between the H atom of the *cis*-thio­amide group and the pyridine N atom. In the crystal structure, inter­molecular bifurcated N—H⋯S and N—H⋯O hydrogen bonds form centrosymmetric tetra­mers extending along the *b* axis.

## Related literature

For general background, see: Aly *et al.* (2007 ▶); Su *et al.* (2006 ▶). For related structures, see: Duque *et al.* (2008 ▶); Corrêa *et al.* (2008 ▶); Theodoro *et al.* (2008 ▶); Valdés-Martínez *et al.* (2002 ▶); Koch (2001 ▶); Pérez *et al.* (2008 ▶). For the synthesis, see: Otazo-Sánchez *et al.* (2001 ▶).
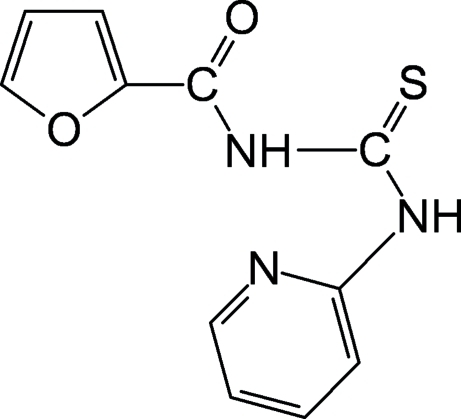


## Experimental

### 

#### Crystal data

C_11_H_9_N_3_O_2_S
*M* *_r_* = 247.27
Monoclinic, 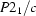

*a* = 6.9510 (1) Å
*b* = 15.7000 (4) Å
*c* = 20.2700 (6) Å
β = 90.284 (2)°
*V* = 2212.05 (9) Å^3^
*Z* = 8
Mo *K*α radiation
μ = 0.29 mm^−1^
*T* = 150 K
0.12 × 0.08 × 0.06 mm

#### Data collection

Enraf–Nonius KappaCCD diffractometer
Absorption correction: none
22281 measured reflections
4337 independent reflections
3574 reflections with *I* > 2σ(*I*)
*R*_int_ = 0.060

#### Refinement

*R*[*F* ^2^ > 2σ(*F* ^2^)] = 0.042
*wR*(*F* ^2^) = 0.122
*S* = 1.10
4337 reflections
323 parameters
H atoms treated by a mixture of independent and constrained refinement
Δρ_max_ = 0.45 e Å^−3^
Δρ_min_ = −0.46 e Å^−3^

### 

Data collection: *COLLECT* (Nonius, 2000 ▶); cell refinement: *SCALEPACK* (Otwinowski & Minor, 1997 ▶); data reduction: *DENZO* (Otwinowski & Minor, 1997 ▶) and *SCALEPACK*; program(s) used to solve structure: *SHELXS97* (Sheldrick, 2008 ▶); program(s) used to refine structure: *SHELXL97* (Sheldrick, 2008 ▶); molecular graphics: *ORTEP-3 for Windows* (Farrugia, 1997 ▶) and *Mercury* (Macrae *et al.*, 2006 ▶); software used to prepare material for publication: *WinGX* (Farrugia, 1999 ▶).

## Supplementary Material

Crystal structure: contains datablocks global, I. DOI: 10.1107/S1600536809011301/ng2563sup1.cif

Structure factors: contains datablocks I. DOI: 10.1107/S1600536809011301/ng2563Isup2.hkl

Additional supplementary materials:  crystallographic information; 3D view; checkCIF report

## Figures and Tables

**Table 1:** Hydrogen-bond geometry (Å, °)

*D*—H⋯*A*	*D*—H	H⋯*A*	*D*⋯*A*	*D*—H⋯*A*
N1—H1⋯O2	0.89 (2)	2.23 (2)	2.653 (2)	109.3 (18)
N1—H1⋯N3	0.89 (2)	1.84 (2)	2.612 (2)	145 (2)
N1*A*—H1*A*⋯O2*A*	0.87 (2)	2.22 (2)	2.661 (2)	111.6 (18)
N1*A*—H1*A*⋯N3*A*	0.87 (2)	1.90 (2)	2.632 (2)	141 (2)
N2—H2⋯O1*A*^i^	0.84 (3)	2.13 (2)	2.940 (2)	162 (2)
N2*A*—H2*A*⋯S1*A*^i^	0.86 (2)	2.51 (2)	3.3530 (15)	170 (2)

Symmetry code: (i) 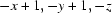.

**Fig. 1.**
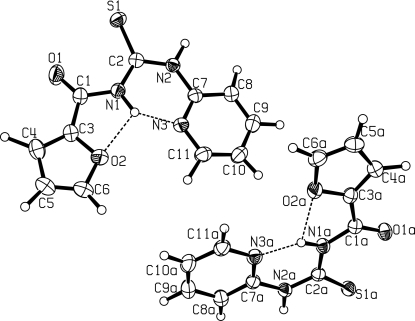
The molecular structure of the title compound. Displacement ellipsoids are drawn at the 50% probability level. The intramolecular N—H···O hydrogen bond is shown as a dashed line.

**Fig. 2.**
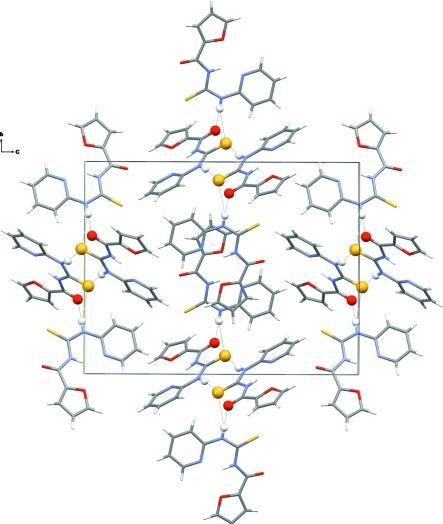
View of the crystal packing of the title compound. Intermolecular hydrogen bonds are shown as dashed lines.

## supplementary crystallographic information

### Comment

The importance of aroylthioureas is found largely in heterocyclic syntheses and many of these substrates have interesting biological activities (Aly *et al.*, 2007). Aroylthioureas have also attracted much attention because of their unique properties, such as the strong coordination ability (Su *et al.*, 2006). The title compound (I), Fig. 1, was synthesized from furoyl isothiocyanate and 2-aminopyridine in dry acetone. Studies of a number of substituted thioureas, including *N*-furoylthioureas, show intramolecular hydrogen bonding between N'H and the furoyl oxygen (Duque *et al.*, 2008; Theodoro *et al.*, 2008; Corrêa *et al.*, 2008). There is also an intermolecular NH hydrogen bond with a sulfur of a neighboring molecule to form a two-dimensional network in these latter thioureas. The molecule structure of the title compound is shown in Figure 1. This thiourea derivative, like other pyridyl thioureas, is found in a conformation resulting from intramolecular hydrogen bonding of N2H(N'H) to the pyridine nitrogen, N3, and *cis*-*cis* like *N*-phenyl- *N*'-(2-pyridyl)thiourea derivatives (Valdés-Martínez *et al.*, 2002). The title compound crystallizes in the thioamide form with two independent molecules in the asymmetric unit. The main bond lengths are within the ranges obtained for similar compounds (Koch *et al.*, 2001 and Pérez *et al.* 2008). The C2—S1 and C1—O1 bonds (Table 1) both show the expected double-bond character. The short values of the C2—N1, C2—N2 and C1—N2 bonds indicate partial double bond character. These results can be explained by the existence of resonance in this part of the molecule. The C=S distance for compound I (two unique molecules) averages 1.667 (2) Å. The furan carbonyl (O1—C1—C3—O2 and O1a—C1a—C3a—O2a, two unique molecules) groups are inclined at an angle of -3.3 (3) ° and 0.6 (3) ° with respect to the plane formed by the thiourea moiety, whereas the 2-pyridyl (C7—C8—C9—C10—C11 and C7a—C8a—C9a—C10*a*—C11*a*, two unique molecules) rings are inclined at an angle of -3.3 (3) ° and 0.6 (3) °, respectively. In addition, the dihedral angles of two independent molecules between the furoyl groups and pyridine ring planes are 85.1 (2)° and 82.96 (8) °, respectively. The *trans*-*cis* geometry in the thiourea moiety is stabilized by the N1—H1···N3 intramolecular hydrogen bond. Another weaker bifurcated intramolecular hydrogen interaction between the furan oxygen atom O2 and the N1—H1 hydrogen atom is observed. The crystal structure is very interesting, stabilized by intermolecular bifurcated N—H···S (non bonding distance of 3.353 (2) Å and bond angle of 170 (2)°) and N—H···O (non bonding distance of 2.940 (2) Å and bond angle of 162 (2)°) hydrogen bonds forming centrosymmetric tetramers extending along the *b* axis.

### Experimental

The title compound (I) was synthesized according to a previous report (Otazo-Sánchez *et al.*, 2001), by converting furoyl chloride into furoyl isothiocyanate and then condensing with 2-aminopyridine. The resulting solid product was crystallized from ethanol yielding X-ray quality single crystals (m.p 150–151 °C). Elemental analysis (%) for C_11_H_9_N_3_O_2_S calculated: C 53.44, H 3.64, N 17.00, S 12.96; found: C 53.50, H 3.46, N 16.99, S 12.58.

### Refinement

H atoms on the C atoms were positioned geometrically with C—H = 0.93–0.97 Å and constrained to ride on their parent atoms with *U*_iso_(H)=1.2*U*_eq_(parent atom).

### Crystal data

**Table:**

C_11_H_9_N_3_O_2_S	*F*(000) = 1024
*M**_r_* = 247.27	*D*_x_ = 1.485 Mg m^−^^3^
Monoclinic, *P*2_1_/*c*	Mo *K*α radiation, λ = 0.71073 Å
Hall symbol: -P 2ybc	Cell parameters from 13181 reflections
*a* = 6.9510 (1) Å	θ = 2.9–26.0°
*b* = 15.7000 (4) Å	µ = 0.28 mm^−^^1^
*c* = 20.2700 (6) Å	*T* = 150 K
β = 90.284 (2)°	Block, colorless
*V* = 2212.05 (9) Å^3^	0.12 × 0.08 × 0.06 mm
*Z* = 8	

### Data collection

**Table:**

Enraf–Nonius KappaCCD diffractometer	3574 reflections with *I* > 2σ(*I*)
Radiation source: fine-focus sealed tube Enraf Nonius FR590	*R*_int_ = 0.060
horizontally mounted graphite crystal	θ_max_ = 26.0°, θ_min_ = 2.9°
φ scans and ω scans with κ offsets	*h* = −8→8
22281 measured reflections	*k* = −19→18
4337 independent reflections	*l* = −24→24

### Refinement

**Table:**

Refinement on *F*^2^	0 restraints
Least-squares matrix: full	H atoms treated by a mixture of independent and constrained refinement
*R*[*F*^2^ > 2σ(*F*^2^)] = 0.042	*w* = 1/[σ^2^(*F*_o_^2^) + (0.0699*P*)^2^ + 0.3338*P*] where *P* = (*F*_o_^2^ + 2*F*_c_^2^)/3
*wR*(*F*^2^) = 0.122	(Δ/σ)_max_ = 0.001
*S* = 1.10	Δρ_max_ = 0.45 e Å^−^^3^
4337 reflections	Δρ_min_ = −0.46 e Å^−^^3^
323 parameters	

### Special details

**Table:**

Geometry. All e.s.d.'s (except the e.s.d. in the dihedral angle between two l.s. planes) are estimated using the full covariance matrix. The cell e.s.d.'s are taken into account individually in the estimation of e.s.d.'s in distances, angles and torsion angles; correlations between e.s.d.'s in cell parameters are only used when they are defined by crystal symmetry. An approximate (isotropic) treatment of cell e.s.d.'s is used for estimating e.s.d.'s involving l.s. planes.

### Fractional atomic coordinates and isotropic or equivalent isotropic displacement parameters (Å^2^)

**Table:**

	*x*	*y*	*z*	*U*_iso_*/*U*_eq_	
S1A	0.26900 (7)	0.58701 (3)	−0.01361 (2)	0.03185 (16)	
S1	0.86024 (7)	0.20621 (3)	−0.13099 (2)	0.03742 (17)	
N1A	0.0427 (2)	0.55696 (11)	0.09207 (8)	0.0285 (4)	
O1A	−0.12681 (18)	0.64878 (9)	0.02319 (7)	0.0327 (3)	
O2	0.7008 (2)	−0.07818 (9)	0.00044 (7)	0.0379 (4)	
N3	0.8621 (2)	0.08935 (10)	0.07607 (8)	0.0313 (4)	
N2A	0.3235 (2)	0.48042 (10)	0.08445 (8)	0.0292 (4)	
N1	0.7790 (2)	0.07419 (10)	−0.04921 (9)	0.0297 (4)	
O2A	−0.23343 (19)	0.56744 (9)	0.18216 (7)	0.0370 (3)	
N2	0.8903 (2)	0.19839 (11)	−0.00248 (8)	0.0278 (4)	
C3	0.6597 (3)	−0.06607 (12)	−0.06512 (10)	0.0311 (4)	
C2A	0.2051 (2)	0.53986 (12)	0.05684 (9)	0.0268 (4)	
C1	0.6980 (3)	0.01830 (13)	−0.09411 (10)	0.0330 (4)	
C11	0.8702 (3)	0.06331 (14)	0.13915 (10)	0.0370 (5)	
H11	0.8452	0.0063	0.1483	0.044*	
N3A	0.1495 (2)	0.44154 (11)	0.17897 (8)	0.0316 (4)	
C1A	−0.1108 (3)	0.60931 (12)	0.07437 (9)	0.0277 (4)	
C11A	0.1415 (3)	0.40025 (13)	0.23715 (10)	0.0358 (5)	
H11A	0.0287	0.4035	0.2615	0.043*	
C7A	0.3113 (3)	0.43617 (12)	0.14412 (10)	0.0294 (4)	
C7	0.8961 (2)	0.17098 (12)	0.06335 (9)	0.0270 (4)	
O1	0.6582 (3)	0.03377 (10)	−0.15091 (8)	0.0527 (4)	
C8	0.9405 (3)	0.23029 (14)	0.11254 (10)	0.0340 (4)	
H8	0.9641	0.2871	0.1022	0.041*	
C3A	−0.2598 (3)	0.61301 (12)	0.12527 (9)	0.0290 (4)	
C6A	−0.3910 (3)	0.58333 (14)	0.22036 (11)	0.0404 (5)	
H6A	−0.4116	0.5604	0.262	0.048*	
C10A	0.2925 (3)	0.35352 (14)	0.26215 (11)	0.0411 (5)	
H10A	0.2823	0.3257	0.3025	0.049*	
C4A	−0.4268 (3)	0.65642 (12)	0.12727 (10)	0.0312 (4)	
H4A	−0.4767	0.6921	0.0948	0.037*	
C6	0.6596 (3)	−0.16117 (14)	0.01396 (12)	0.0418 (5)	
H6	0.6744	−0.1867	0.0551	0.05*	
C9	0.9482 (3)	0.20191 (14)	0.17660 (11)	0.0401 (5)	
H9	0.9766	0.2398	0.2105	0.048*	
C2	0.8401 (2)	0.15650 (12)	−0.05889 (9)	0.0275 (4)	
C5A	−0.5112 (3)	0.63660 (14)	0.18931 (11)	0.0368 (5)	
H5A	−0.6279	0.6569	0.2052	0.044*	
C10	0.9135 (3)	0.11655 (15)	0.19077 (10)	0.0389 (5)	
H10	0.9195	0.0963	0.2338	0.047*	
C9A	0.4598 (3)	0.34887 (15)	0.22583 (11)	0.0448 (5)	
H9A	0.5645	0.3182	0.2418	0.054*	
C4	0.5942 (3)	−0.13956 (14)	−0.09150 (12)	0.0410 (5)	
H4	0.5561	−0.1485	−0.135	0.049*	
C8A	0.4710 (3)	0.38987 (14)	0.16572 (10)	0.0379 (5)	
H8A	0.5818	0.3867	0.1403	0.045*	
C5	0.5951 (3)	−0.20049 (14)	−0.03991 (12)	0.0410 (5)	
H5	0.5577	−0.2572	−0.0431	0.049*	
H2A	0.425 (3)	0.4683 (14)	0.0623 (11)	0.037 (6)*	
H2	0.934 (3)	0.2477 (17)	−0.0082 (12)	0.044 (7)*	
H1A	0.024 (3)	0.5257 (16)	0.1268 (12)	0.043 (6)*	
H1	0.796 (3)	0.0567 (15)	−0.0081 (12)	0.041 (6)*	

### Atomic displacement parameters (Å^2^)

**Table:**

	*U*^11^	*U*^22^	*U*^33^	*U*^12^	*U*^13^	*U*^23^
S1A	0.0344 (3)	0.0315 (3)	0.0297 (3)	0.00331 (19)	0.00427 (19)	0.0052 (2)
S1	0.0482 (3)	0.0361 (3)	0.0280 (3)	−0.0017 (2)	0.0017 (2)	0.0040 (2)
N1A	0.0284 (8)	0.0290 (9)	0.0281 (9)	0.0000 (6)	0.0010 (6)	0.0050 (7)
O1A	0.0311 (7)	0.0331 (7)	0.0338 (8)	−0.0008 (6)	−0.0009 (5)	0.0078 (6)
O2	0.0430 (8)	0.0330 (8)	0.0377 (9)	−0.0026 (6)	0.0002 (6)	0.0033 (6)
N3	0.0331 (8)	0.0301 (9)	0.0308 (9)	−0.0014 (7)	−0.0010 (6)	0.0036 (7)
N2A	0.0291 (8)	0.0297 (9)	0.0287 (9)	0.0039 (7)	0.0026 (6)	0.0016 (7)
N1	0.0338 (9)	0.0277 (9)	0.0277 (9)	0.0000 (6)	−0.0026 (7)	0.0006 (7)
O2A	0.0383 (8)	0.0430 (9)	0.0298 (8)	0.0064 (6)	0.0011 (6)	0.0052 (6)
N2	0.0289 (8)	0.0251 (9)	0.0294 (9)	−0.0025 (7)	0.0005 (6)	0.0012 (7)
C3	0.0282 (9)	0.0312 (10)	0.0340 (11)	0.0029 (8)	−0.0028 (7)	−0.0032 (8)
C2A	0.0285 (9)	0.0236 (10)	0.0282 (10)	−0.0023 (7)	−0.0014 (7)	−0.0019 (7)
C1	0.0354 (10)	0.0309 (11)	0.0327 (12)	0.0022 (8)	−0.0039 (8)	−0.0034 (8)
C11	0.0377 (11)	0.0383 (12)	0.0351 (12)	0.0001 (9)	−0.0003 (8)	0.0087 (9)
N3A	0.0361 (9)	0.0301 (9)	0.0285 (9)	−0.0011 (7)	0.0021 (6)	0.0014 (7)
C1A	0.0275 (9)	0.0254 (10)	0.0302 (11)	−0.0045 (7)	−0.0026 (7)	0.0012 (8)
C11A	0.0466 (12)	0.0326 (11)	0.0283 (11)	−0.0042 (9)	0.0026 (8)	0.0012 (8)
C7A	0.0359 (10)	0.0242 (9)	0.0280 (10)	−0.0011 (8)	−0.0016 (8)	0.0002 (8)
C7	0.0216 (8)	0.0302 (10)	0.0293 (10)	0.0009 (7)	0.0011 (7)	0.0023 (8)
O1	0.0830 (12)	0.0382 (9)	0.0368 (9)	−0.0051 (8)	−0.0204 (8)	−0.0002 (7)
C8	0.0349 (10)	0.0325 (11)	0.0344 (11)	−0.0032 (8)	−0.0006 (8)	−0.0009 (9)
C3A	0.0313 (10)	0.0280 (10)	0.0277 (10)	−0.0029 (8)	−0.0026 (7)	0.0016 (8)
C6A	0.0450 (12)	0.0450 (13)	0.0312 (12)	−0.0007 (9)	0.0083 (9)	−0.0009 (9)
C10A	0.0553 (13)	0.0387 (12)	0.0294 (11)	0.0014 (10)	−0.0013 (9)	0.0083 (9)
C4A	0.0301 (10)	0.0285 (10)	0.0349 (11)	−0.0014 (8)	−0.0021 (7)	0.0028 (8)
C6	0.0418 (12)	0.0326 (12)	0.0510 (14)	−0.0034 (9)	0.0031 (9)	0.0079 (10)
C9	0.0415 (11)	0.0460 (13)	0.0326 (12)	0.0006 (9)	−0.0045 (8)	−0.0061 (10)
C2	0.0238 (8)	0.0273 (10)	0.0314 (11)	0.0044 (7)	0.0018 (7)	−0.0007 (8)
C5A	0.0322 (10)	0.0382 (12)	0.0401 (12)	−0.0015 (9)	0.0065 (8)	−0.0052 (9)
C10	0.0368 (11)	0.0523 (14)	0.0276 (11)	0.0016 (9)	−0.0003 (8)	0.0046 (10)
C9A	0.0500 (13)	0.0462 (13)	0.0383 (13)	0.0116 (10)	−0.0053 (10)	0.0099 (10)
C4	0.0341 (11)	0.0399 (12)	0.0489 (14)	0.0018 (9)	−0.0076 (9)	−0.0103 (10)
C8A	0.0394 (11)	0.0395 (12)	0.0348 (12)	0.0068 (9)	0.0004 (8)	0.0040 (9)
C5	0.0323 (11)	0.0302 (11)	0.0604 (15)	−0.0014 (8)	−0.0008 (9)	0.0028 (10)

### Geometric parameters (Å, °)

**Table:**

S1A—C2A	1.6705 (19)	N3A—C11A	1.347 (3)
S1—C2	1.6633 (19)	C1A—C3A	1.467 (3)
N1A—C2A	1.365 (2)	C11A—C10A	1.375 (3)
N1A—C1A	1.393 (2)	C11A—H11A	0.93
N1A—H1A	0.87 (2)	C7A—C8A	1.395 (3)
O1A—C1A	1.213 (2)	C7—C8	1.398 (3)
O2—C6	1.362 (3)	C8—C9	1.373 (3)
O2—C3	1.371 (2)	C8—H8	0.93
N3—C7	1.329 (2)	C3A—C4A	1.346 (3)
N3—C11	1.343 (3)	C6A—C5A	1.338 (3)
N2A—C2A	1.363 (2)	C6A—H6A	0.93
N2A—C7A	1.398 (3)	C10A—C9A	1.381 (3)
N2A—H2A	0.86 (2)	C10A—H10A	0.93
N1—C2	1.375 (3)	C4A—C5A	1.425 (3)
N1—C1	1.382 (2)	C4A—H4A	0.93
N1—H1	0.89 (2)	C6—C5	1.330 (3)
O2A—C6A	1.367 (3)	C6—H6	0.93
O2A—C3A	1.369 (2)	C9—C10	1.392 (3)
N2—C2	1.363 (2)	C9—H9	0.93
N2—C7	1.402 (2)	C5A—H5A	0.93
N2—H2	0.84 (3)	C10—H10	0.93
C3—C4	1.350 (3)	C9A—C8A	1.381 (3)
C3—C1	1.474 (3)	C9A—H9A	0.93
C1—O1	1.207 (2)	C4—C5	1.417 (3)
C11—C10	1.371 (3)	C4—H4	0.93
C11—H11	0.93	C8A—H8A	0.93
N3A—C7A	1.334 (2)	C5—H5	0.93
			
C2A—N1A—C1A	128.01 (17)	C9—C8—H8	121.1
C2A—N1A—H1A	116.0 (15)	C7—C8—H8	121.1
C1A—N1A—H1A	115.3 (15)	C4A—C3A—O2A	110.57 (17)
C6—O2—C3	106.52 (16)	C4A—C3A—C1A	130.76 (18)
C7—N3—C11	118.14 (18)	O2A—C3A—C1A	118.66 (16)
C2A—N2A—C7A	131.03 (17)	C5A—C6A—O2A	110.37 (19)
C2A—N2A—H2A	115.6 (15)	C5A—C6A—H6A	124.8
C7A—N2A—H2A	113.4 (15)	O2A—C6A—H6A	124.8
C2—N1—C1	128.90 (18)	C11A—C10A—C9A	118.3 (2)
C2—N1—H1	112.8 (15)	C11A—C10A—H10A	120.8
C1—N1—H1	118.3 (15)	C9A—C10A—H10A	120.8
C6A—O2A—C3A	106.10 (15)	C3A—C4A—C5A	105.96 (18)
C2—N2—C7	131.01 (17)	C3A—C4A—H4A	127
C2—N2—H2	114.8 (16)	C5A—C4A—H4A	127
C7—N2—H2	114.0 (16)	C5—C6—O2	110.4 (2)
C4—C3—O2	109.50 (18)	C5—C6—H6	124.8
C4—C3—C1	132.2 (2)	O2—C6—H6	124.8
O2—C3—C1	118.28 (17)	C8—C9—C10	120.1 (2)
N2A—C2A—N1A	114.79 (17)	C8—C9—H9	119.9
N2A—C2A—S1A	119.45 (14)	C10—C9—H9	119.9
N1A—C2A—S1A	125.73 (14)	N2—C2—N1	114.32 (17)
O1—C1—N1	126.22 (19)	N2—C2—S1	119.24 (15)
O1—C1—C3	121.33 (18)	N1—C2—S1	126.44 (15)
N1—C1—C3	112.44 (17)	C6A—C5A—C4A	107.00 (18)
N3—C11—C10	123.3 (2)	C6A—C5A—H5A	126.5
N3—C11—H11	118.4	C4A—C5A—H5A	126.5
C10—C11—H11	118.4	C11—C10—C9	117.83 (19)
C7A—N3A—C11A	118.13 (17)	C11—C10—H10	121.1
O1A—C1A—N1A	126.07 (17)	C9—C10—H10	121.1
O1A—C1A—C3A	121.29 (17)	C10A—C9A—C8A	119.8 (2)
N1A—C1A—C3A	112.65 (16)	C10A—C9A—H9A	120.1
N3A—C11A—C10A	123.00 (19)	C8A—C9A—H9A	120.1
N3A—C11A—H11A	118.5	C3—C4—C5	106.5 (2)
C10A—C11A—H11A	118.5	C3—C4—H4	126.7
N3A—C7A—C8A	122.58 (18)	C5—C4—H4	126.7
N3A—C7A—N2A	118.80 (17)	C9A—C8A—C7A	118.12 (19)
C8A—C7A—N2A	118.60 (17)	C9A—C8A—H8A	120.9
N3—C7—C8	122.89 (18)	C7A—C8A—H8A	120.9
N3—C7—N2	118.45 (17)	C6—C5—C4	107.01 (19)
C8—C7—N2	118.65 (17)	C6—C5—H5	126.5
C9—C8—C7	117.75 (19)	C4—C5—H5	126.5
			
C6—O2—C3—C4	0.1 (2)	C6A—O2A—C3A—C1A	179.28 (17)
C6—O2—C3—C1	−177.81 (17)	O1A—C1A—C3A—C4A	−0.9 (3)
C7A—N2A—C2A—N1A	−2.9 (3)	N1A—C1A—C3A—C4A	179.17 (19)
C7A—N2A—C2A—S1A	175.31 (16)	O1A—C1A—C3A—O2A	−179.68 (17)
C1A—N1A—C2A—N2A	−174.81 (17)	N1A—C1A—C3A—O2A	0.4 (2)
C1A—N1A—C2A—S1A	7.1 (3)	C3A—O2A—C6A—C5A	−0.2 (2)
C2—N1—C1—O1	−3.4 (3)	N3A—C11A—C10A—C9A	0.0 (3)
C2—N1—C1—C3	177.41 (17)	O2A—C3A—C4A—C5A	−0.3 (2)
C4—C3—C1—O1	5.4 (3)	C1A—C3A—C4A—C5A	−179.11 (19)
O2—C3—C1—O1	−177.24 (19)	C3—O2—C6—C5	−0.1 (2)
C4—C3—C1—N1	−175.4 (2)	C7—C8—C9—C10	0.4 (3)
O2—C3—C1—N1	2.0 (2)	C7—N2—C2—N1	2.3 (3)
C7—N3—C11—C10	−0.6 (3)	C7—N2—C2—S1	−176.68 (14)
C2A—N1A—C1A—O1A	0.7 (3)	C1—N1—C2—N2	171.78 (17)
C2A—N1A—C1A—C3A	−179.38 (17)	C1—N1—C2—S1	−9.3 (3)
C7A—N3A—C11A—C10A	0.4 (3)	O2A—C6A—C5A—C4A	0.0 (2)
C11A—N3A—C7A—C8A	−0.1 (3)	C3A—C4A—C5A—C6A	0.1 (2)
C11A—N3A—C7A—N2A	−178.41 (17)	N3—C11—C10—C9	0.8 (3)
C2A—N2A—C7A—N3A	9.8 (3)	C8—C9—C10—C11	−0.7 (3)
C2A—N2A—C7A—C8A	−168.59 (19)	C11A—C10A—C9A—C8A	−0.7 (4)
C11—N3—C7—C8	0.3 (3)	O2—C3—C4—C5	−0.2 (2)
C11—N3—C7—N2	179.18 (16)	C1—C3—C4—C5	177.4 (2)
C2—N2—C7—N3	5.8 (3)	C10A—C9A—C8A—C7A	0.9 (3)
C2—N2—C7—C8	−175.35 (17)	N3A—C7A—C8A—C9A	−0.5 (3)
N3—C7—C8—C9	−0.2 (3)	N2A—C7A—C8A—C9A	177.75 (19)
N2—C7—C8—C9	−179.06 (16)	O2—C6—C5—C4	0.0 (2)
C6A—O2A—C3A—C4A	0.3 (2)	C3—C4—C5—C6	0.1 (2)

### Hydrogen-bond geometry (Å, °)

**Table:**

*D*—H···*A*	*D*—H	H···*A*	*D*···*A*	*D*—H···*A*
N1—H1···O2	0.89 (2)	2.23 (2)	2.653 (2)	109.3 (18)
N1—H1···N3	0.89 (2)	1.84 (2)	2.612 (2)	145 (2)
N1A—H1A···O2A	0.87 (2)	2.22 (2)	2.661 (2)	111.6 (18)
N1A—H1A···N3A	0.87 (2)	1.90 (2)	2.632 (2)	141 (2)
N2—H2···O1A^i^	0.84 (3)	2.13 (2)	2.940 (2)	162 (2)
N2A—H2A···S1A^i^	0.86 (2)	2.51 (2)	3.3530 (15)	170 (2)

Symmetry codes: (i) −*x*+1, −*y*+1, −*z*.
